# A long non‐coding RNA signature for diagnostic prediction of sepsis upon ICU admission

**DOI:** 10.1002/ctm2.123

**Published:** 2020-07-02

**Authors:** Xueyan Liu, Xubin Zheng, Jun Wang, Ning Zhang, Kwong‐Sak Leung, Xiufeng Ye, Lixin Cheng

**Affiliations:** ^1^ Department of Critical Care Medicine Shenzhen People's Hospital First Affiliated Hospital of Southern University of Science and Technology Shenzhen China; ^2^ Shenzhen People's Hospital First Affiliated Hospital of Southern University of Science and Technology Shenzhen China; ^3^ Department of Computer Science and Engineering The Chinese University of Hong Kong Shatin New Territories Hong Kong

Dear Editor,

Sepsis, the highest mortality disease in critically ill patients, is clinically diagnosed through the dysregulated systemic inflammatory response of patients to infection in the presence of organ dysfunction.^1^, ^2^, ^3^ No effective biomarkers and approved molecular therapies have been developed for sepsis to diagnose and treat the immune response state of the patients, leading to the management of these critically ill patients only relies on early recognition by experience and supportive care.^4^, ^5^ Long noncoding RNAs (lncRNAs) are implicated in a wide variety of biological processes and accumulative studies have demonstrated that several dysregulated lncRNAs play important roles in tumorigenesis and tumor progression.^6^, ^7^, ^8^ However, the lncRNA signature has not been studied for the rapid diagnosis of sepsis, due to the limitation of data sources and lack of RNA‐seq datasets.^3^ Hence, we analyzed three whole blood transcriptome cohorts of critically ill adult patients and identified a 28‐lncRNA signature for sepsis diagnosis, which imputes a score to assess the risk of sepsis.

The expression profiling of 3745 lncRNAs in three cohorts, GSE95233, GSE28750, and GSE57065, were normalized and reannotated for the investigation^6^, ^9^ (Table S1). The largest cohort GSE95233 was set as the discovery dataset, while the other two independent cohorts were set as the validation datasets. To select lncRNAs for the predictive signature, we first determined 84 differentially expressed (DE) lncRNAs between sepsis patients and healthy individuals based on the discovery dataset. Then we took advantage of a regression algorithm least absolute shrinkage and selection operator (LASSO) to further identify 28 predictive lncRNAs, named SepSig28, which serves as a molecular diagnostic signature to calculate the risk score to predict whether individuals were suffering from sepsis or not. After that, we validated the diagnostic signature in two independent datasets and demonstrated the high performance of the 28 lncRNAs in the risk prediction of sepsis (Figure 1A).

**FIGURE 1:**
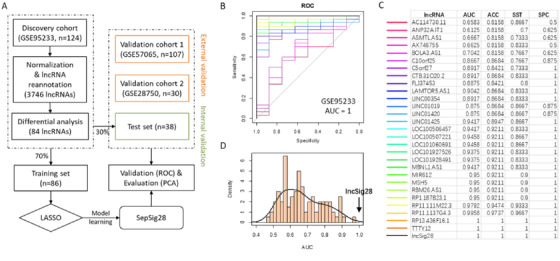
Model construction and internal validation. **A,** Workflow to identify the lncRNA signature of sepsis. **B,** ROC curves for the 28‐lncRNA signature and other 28‐minus‐one lncRNA signatures. **C,** AUC, accuracy, sensitivity, and specificity for the 28‐lncRNA signature and other 28‐minus‐one lncRNA signatures. **D,** Distribution of AUCs for the simulated models in which the lncRNAs were randomly picked up. ROC curve, receiver operating characteristic curve; AUC, area under curve

Risk score = (BOLA3.AS1 × 0.254) + (LINC00354 × 0.1996) + (C5orf27 × 0.1537) + (RP1.187B23.1 × ‐0.1427) + (MBNL1.AS1 × ‐0.1419) + (LINC01420 × ‐0.1140) + (RP13.436F16.1 × 0.1060) + (CTB.31O20.2 × 0.1023) + (LINC01425 × 0.0949) + (C10orf25 × ‐0.0763) + (RP11.111M22.3 × 0.0743) + (LAMTOR5.AS1 × 0.0739) + (FLJ37453 × 0.0713) + (AX746755 × ‐0.0690) + (TTTY12 × 0.0678) + (ASMTL.AS1 × ‐0.0535) + (LOC101928491 × 0.0461) + (RBM26.AS1 × ‐0.0438) + (ANP32A.IT1 × 0.0437) + (LOC101060691 × 0.0319) + (MSH5 × ‐0.0311) + (LOC100507221 × 0.0289) + (RP11.1137G4.3 × ‐0.0245) + (LOC100506457 × 0.0237) + (MIR612 × ‐0.0189) + (AC114730.11 × 0.0079) + (LOC101927526 × 0.0026) + (LINC01019 × ‐0.0020). The values following the symbols are the importance weights of the expression abundance of each lncRNA. These lncRNAs are listed in order of decreasing importance.

When tuned in the discovery dataset using fivefold cross‐validation, the SepSig28 can perfectly classify the sepsis patient samples and healthy control samples, with all the measures equal 1, including the area under curve (AUC), accuracy, sensitivity, and specificity (Figure 1B,C). To test the randomness of the model, we randomly picked up an equivalent number of lncRNAs 1000 times and evaluated their performance using the same procedure as SepSig28. Our result shows that no random combinations can achieve the score of AUC as high as 1 (Figure 1D). Besides, we constructed all possible 27‐lncRNA signatures (28 minus 1) by excluding one lncRNA once a time to evaluate the predictive capability of each lncRNA in the SepSig28 model. For the discovery dataset, two lncRNA members are not necessary for the model, as the model can perform equally well without either of them (Figure 1C). We added these two as supplementary features to make the model more robust.

In the independent cohorts GSE28750 and GSE57065, the hierarchical clustering shows altered expression pattern of the SepSig28 lncRNAs cannot well distinguish sepsis patient samples from the normal ones (Figure 2A,B). Using the computed risk scores by weighted sum, however, SepSig28 can achieve the AUC scores as high as 0.9712 for GSE57065 and 0.95 for GSE28750, respectively (Figure 2C,D), which outperforms almost all the other combinations of 27 (28 minus 1) lncRNAs. Overall, SepSig28 has the best classification performance for all three cohorts according to the measures of AUC, accuracy, sensitivity, and specificity (Figure 2E).

**FIGURE 2:**
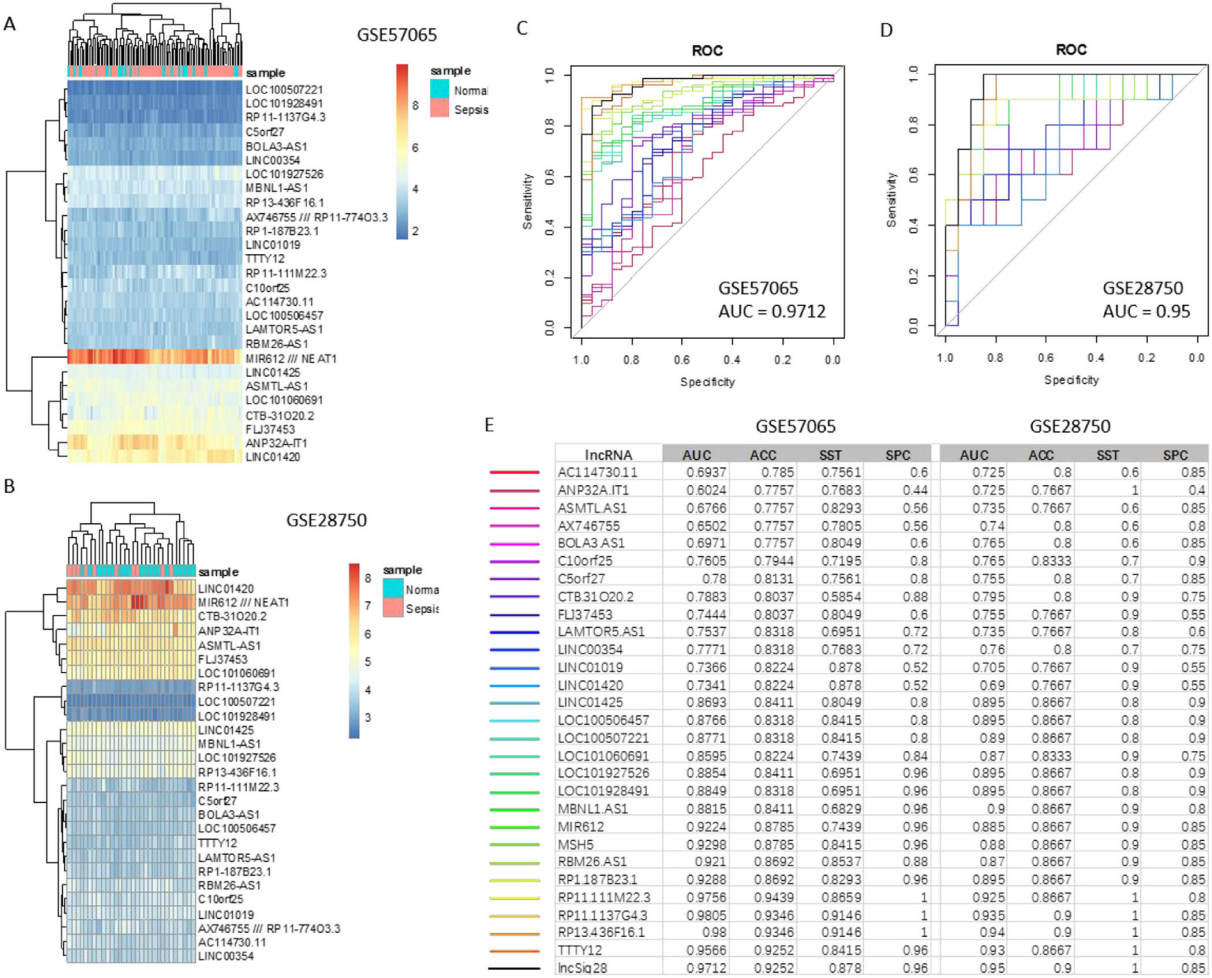
External validation of SepSig28. Hierarchical clustering of the expression samples based on the 28‐lncRNA signature in dataset GSE57065 **(A)** and 28750 **(B)**, respectively. ROC curves for the 28‐lncRNA signature and other 28‐minus‐one lncRNA signatures in dataset GSE57065 **(C)** and 28750 **(D). E,** AUC, accuracy, sensitivity, and specificity for the 28‐lncRNA signature and other 28‐minus‐one lncRNA signatures in the two validation cohorts

To investigate the biological functions the SepSig28 involved, we associated them with their co‐expressed genes across the sepsis samples of each cohort. Genes co‐expressed with the lncRNAs in all the cohorts (Pearson correlation coefficient > 0.7) were considered to be co‐expressed. Gene Ontology (GO) and KEGG pathway enrichment analysis were separately performed for the set of co‐expressed genes.^10^ GO enrichment analysis showed that the lncRNAs of SepSig28 are mainly involved in three biological processes, including hormone mediated signaling pathway, RNA splicing, and histone modification (Figure S1A). KEGG analysis showed the SepSig28 associated genes are significantly implicated in pathways that are known to be related to sepsis pathogenesis, including Wnt signaling pathway, Th17 cell differentiation, Notch signaling pathway, etc. (Figure S1B). Interestingly, both GO and KEGG enrichment revealed that lncRNAs in SepSig28 tend to participate in hormone signaling related pathways, indicating an underlying association between hormone signaling and sepsis.

In conclusion, we identified and validated the first non‐coding signature consisting of 28 lncRNAs that can well distinguish sepsis patients from healthy controls for adults. Despite limitations such as the limited number of lncRNA features and the small sample size, we provided evidence that lncRNAs could be adopted as markers for the diagnosis of critical diseases. The proposed model could be used as an alternative or complementary diagnostic metric for sepsis.

## AUTHOR CONTRIBUTIONS

LC conceived the idea and drafted the manuscript. LC performed data analysis. XL, XZ, JW, NZ, and RW performed data management and analysis. XL, KL, and XY helped interpret the results and give suggestions. All authors read and approved the final manuscript.

## CONFLICT OF INTEREST

The authors declare no conflict of interest.

## Supporting information

Figure S1. Functional analysis of the protein‐coding genes co‐expressed with the 28 lncRNAs in SepSig28. (A) Functional network of the enriched GO terms. Nodes represent enriched GO terms while edges represent Kappa scores among the nodes. Only the edges with Kappa scores over 0.5 are shown. Node size represents the number of coexpressed genes in GO terms, while color indicates the statistical significance of term enrichment. (B) The enriched KEGG pathways. Node size represents the number of coexpressed genes in the pathways, while the color represents the enrichment significance.

Table S1. Discovery and validation cohorts used in this study.
