# Supplementary figures and images for: A long non‐coding RNA signature for diagnostic prediction of sepsis upon ICU admission
Source: Clin Transl Med. 2020 Jul 2;10(3):e123. doi: 10.1002/ctm2.123 (PMC7418814; doi:10.1002/ctm2.123)

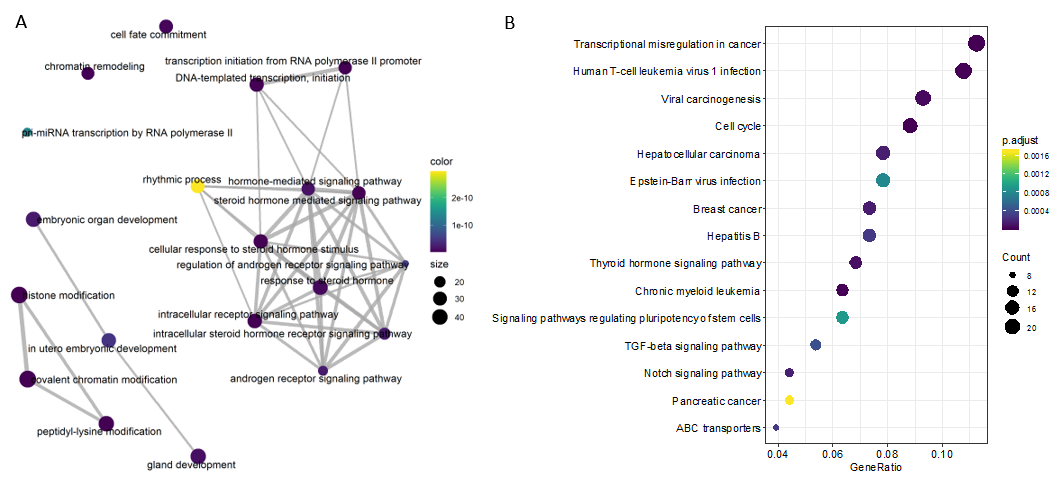

Supplement: Supplementary file 1 — Figure S1. Functional analysis of the protein‐coding genes co‐expressed with the 28 lncRNAs in SepSig28. (A) Functional network of the enriched GO terms. Nodes represent enriched GO terms while edges represent Kappa scores among the nodes. Only the edges with Kappa scores over 0.5 are shown. Node size represents the number of coexpressed genes in GO terms, while color indicates the statistical significance of term enrichment. (B) The enriched KEGG pathways. Node size represents the number of coexpressed genes in the pathways, while the color represents the enrichment significance. [file CTM2-10-e123-s001.tif]
