# Supplementary material for: A long non‐coding RNA signature for diagnostic prediction of sepsis upon ICU admission
Source: Clin Transl Med. 2020 Jul 2;10(3):e123. doi: 10.1002/ctm2.123 (PMC7418814; doi:10.1002/ctm2.123)
Supplement: Supplementary file 2 — Table S1. Discovery and validation cohorts used in this study. [file CTM2-10-e123-s002.pdf]

Table S1. Discovery and validation cohorts used in this study.

GSE Number	Tissue	Sepsis (N)	Control (N)	Platform
GSE95233	Whole blood	Septic shock samples (102)	Healthy controls (22)	HG-U133_Plus_2
GSE57065	Whole blood	Septic shock samples (82)	Healthy controls (25)	HG-U133_Plus_2
GSE28750	Whole blood	Sepsis samples (10)	Healthy controls (20)	HG-U133_Plus_2
